# Association of gut microbiota dietary index with MAFLD and the risk of liver fibrosis: the mediating effect of vitamins

**DOI:** 10.1017/jns.2026.10093

**Published:** 2026-04-13

**Authors:** Jinlu Han, Teng Zhou, Jiong Chen, Ling Xu, Wen Shan, Qinghui Zhang

**Affiliations:** 1 Tongren Hospital Shanghai Jiaotong University School of Medicine, China; 2 Fudan University Shanghai Cancer Center, China; 3 No 2 People’s Hospital of Fuyang City, China

**Keywords:** Dietary Index of Gut Microbiota, liver fibrosis, metabolic-associated fatty liver disease, vitamin mediation, dietary nutrition

## Abstract

Metabolic dysfunction-associated fatty liver disease (MAFLD) is emerging as the leading cause of chronic liver disease worldwide, with a spectrum ranging from simple steatosis to advanced fibrosis and cirrhosis. Its pathogenesis is multifactorial and involves genetic, metabolic, and gut microbiota factors. Gut microbiota, through the gut-liver axis, plays a crucial role in the progression of MAFLD. Here, we investigated the association between DI-GM, a novel metric reflecting diet-microbiota interactions, MAFLD, and liver fibrosis, with a focus on the mediating role of vitamins. Using data from 13,498 participants across seven NHANES cycles (2007–2018), we found that higher DI-GM scores, indicative of a healthier gut microbiota-promoting diet, were associated with a reduced prevalence of MAFLD (OR = 0.93, 95% CI = 0.88–0.99) and high-risk liver fibrosis (OR = 0.94, 95% CI = 0.90–0.98) in fully adjusted models. Notably, the relationship between DI-GM and MAFLD and the risk of liver fibrosis is largely mediated by specific vitamins and carotenoids, with vitamin C and cis-β-carotene emerging as key mediators. These findings suggest that dietary interventions targeting the gut microbiota and vitamin supplementation could offer new strategies for the prevention and management of MAFLD. Our study provides the first comprehensive evidence linking DI-GM to MAFLD and the risk of liver fibrosis, highlighting the potential of diet and nutrition to modulate metabolic liver diseases. Future research should focus on elucidating the underlying mechanisms and validating these findings through prospective studies and clinical trials.

## Introduction

Metabolic dysfunction-associated fatty liver disease (MAFLD), formerly known as non-alcoholic fatty liver disease (NAFLD), is rapidly becoming the leading cause of chronic liver disease worldwide, surpassing the prevalence of viral hepatitis.^(1,2)^ The disease spectrum of MAFLD ranges from simple hepatic steatosis to metabolic dysfunction-associated steatohepatitis (MASH), potentially progressing to liver fibrosis, cirrhosis, and hepatocellular carcinoma.^(3,4)^ The pathogenesis of MAFLD is multifactorial and involves genetic predisposition, insulin resistance, and alterations in the gut microbiota.^(3)^ The gut microbiota plays a crucial role in the onset and progression of MAFLD through the gut-liver axis, where dysbiosis may contribute to the increased transport of bacterial products and metabolites to the liver, thereby promoting inflammation and fibrosis.^(5)^

In recent years, a novel gut microbiota dietary index (DI-GM) has been developed to characterise the dietary components associated with gut microbiota diversity. This index includes beneficial components (e.g., fermented dairy products, whole grains, and fibre) as well as harmful components (e.g., red meat and high-fat diets). Studies suggest that DI-GM may influence the management of MAFLD by modulating gut microbiota diversity and acting through the gut-liver axis.^(6)^ A recent study demonstrated that the gut bacterium *Bacteroides uniformis* can effectively alleviate MASH by producing 3-succinylated bile acid.^(7)^

Additionally, vitamins play a crucial role in liver health, progression of MAFLD and liver fibrosis. Water-soluble vitamins such as vitamin C possess antioxidant properties that help reduce oxidative stress and inflammation, thereby preventing the progression of MAFLD and liver fibrosis.^(8)^ Fat-soluble vitamins, including vitamins A and E, also have significant functions. Vitamin A exhibits antioxidant effects and protects hepatocytes, whereas vitamin E reduces hepatic fat accumulation and improves liver function.^(9)^

This study aimed to investigate the association between liver fibrosis and the newly proposed DI-GM and MAFLD, with a particular focus on the mediating role of vitamin C. By elucidating the interactions among these factors, we hope to provide new insights into the development of dietary interventions targeting the gut microbiota and vitamin C pathways, thereby offering effective strategies for the prevention and management of MAFLD and its complications.

## Methods

### Data source

The data for this study were obtained from publicly available records across six consecutive National Health and Nutrition Examination Survey (NHANES) cycles (2007–2018). The NHANES is an ongoing, cross-sectional study designed to assess the health and nutritional status of a nationally representative, non-institutionalised U.S. population. The study protocol was approved by the Institutional Review Board of the National Center for Health Statistics (NCHS), and informed consent was obtained from all participants. The NHANES employs a complex, multistage probability sampling design to ensure the collection of robust and generalisable data.

### Laboratory measurements

Serum concentrations of vitamins and carotenoids were quantified using standard NHANES laboratory protocols at the CDC’s National Center for Environmental Health. Specifically, vitamin C (ascorbic acid) was measured using isocratic high-performance liquid chromatography (HPLC) with electrochemical detection at 650 mV. Retinol (vitamin A), tocopherols (vitamin E), and carotenoids (including α-carotene, trans-β-carotene, cis-β-carotene, β-cryptoxanthin, lutein/zeaxanthin, and lycopene) were simultaneously measured via HPLC with photodiode array detection, utilising a C18 column for separation. 25-hydroxyvitamin D concentrations (D2 and D3) were determined using ultra-high performance liquid chromatography-tandem mass spectrometry (UHPLC-MS/MS). All assays adhered to rigorous quality control procedures as detailed in the NHANES Laboratory Procedure Manuals.

### Study design and population

**Figure 1.**
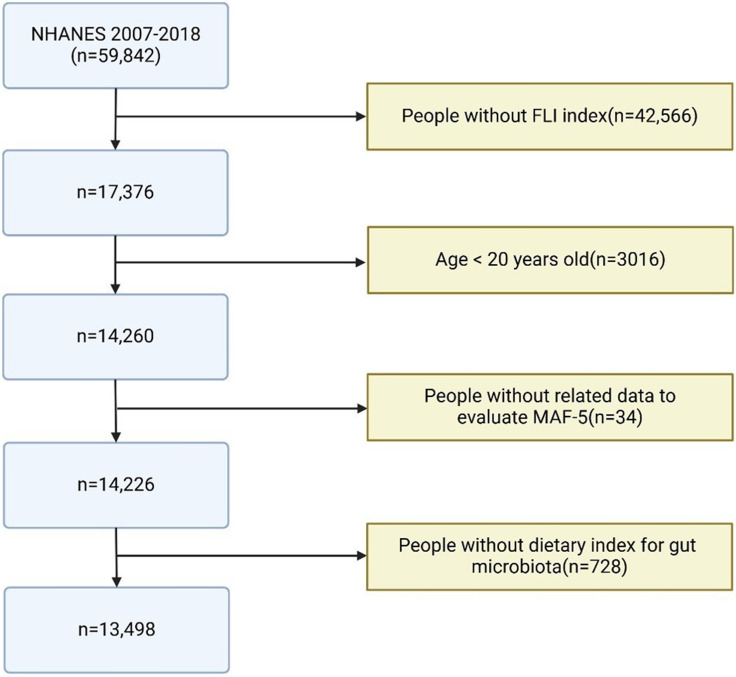
Participant selection flowchart for NHANES 2007–2018 study: Analysis of MAFLD and liver fibrosis.

A total of 59,842 participants were included in this study, spanning from 2007 to 2018, as DI-GM data were only available during this period. The exclusion criteria for the analysis were as follows: missing FLI index data (*n* = 42,466), participants aged under 20 years (*n* = 3016), missing Metabolic Dysfunction–Associated Fibrosis 5 (MAF-5) components data (*n* = 34), and missing DI-GM components data (*n* = 728). As shown in Figure 1, the final analysis included 13,498 eligible participants.^(10)^

### MAFLD diagnosis and MAF-5 score evaluation

We first calculated the US-FLI index for each participant and defined fatty liver (SLD) as a US-FLI ≥ 30.^(11)^ MAFLD is was diagnosed based on the presence of SLD in conjunction with overweight/obesity (body mass index (BMI) ≥ 25 kg/m^2^), type 2 diabetes (T2 DM), or metabolic dysfunction.^(12)^ Metabolic dysfunction was defined by meeting at least two of the following metabolic risk abnormalities: (1) waist circumference ≥ 102 cm in men and ≥88 cm in women; (2) blood pressure ≥ 130/85 mmHg; (3) plasma triglycerides ≥ 150 mg/dl; (4) plasma high-density lipoprotein cholesterol (HDL-C) < 40 mg/dl in men and < 50 mg/dl in women; (5) prediabetes (i.e., fasting glucose levels between 100 and 125 mg/dl, 2-hour post-load glucose levels between 140 and 199 mg/dl, or HbA1c between 5.7% and 6.4%); (6) Homeostasis Model Assessment for Insulin Resistance (HOMA-IR) score ≥ 2.5; 7) plasma high-sensitivity C-reactive protein (hs-CRP) level > 2 mg/l. T2 DM was defined based on the following criteria of the American Diabetes Association: (1) self-reported physician diagnosis of diabetes, (2) use of oral hypoglycaemic agents or insulin treatment, (3) fasting plasma glucose ≥ 126 mg/dl, (4) oral glucose tolerance test ≥ 200 mg/dl, and (5) HbA1c ≥ 6.5%.^(13)^

The MAF-5 score was calculated based on waist circumference, BMI, aspartate aminotransferase (AST) level, platelet count, and presence of diabetes.^(14)^ We defined participants with MAF-5 scores < 1 as the low-risk group, and those with MAF-5 scores ≥ 1 as the high-risk group. In the baseline analysis, participants with both MAFLD and MAF-5 ≥ 1 were categorised into the high-risk group for liver fibrosis. Subsequent regression analyses treated MAFLD and MAF-5 as the independent variables.

### Gut microbiota dietary index assessment

According to the scoring criteria outlined by Kase et al.^(6)^ 14 food items or nutrients were identified as components of DI-GM. These included beneficial components such as avocado, broccoli, chickpeas, coffee, cranberries, fermented dairy products, fibre, green tea (which could not be assessed because of the lack of specific tea type data in NHANES), soy, and whole grains, whereas detrimental components such as red meat, processed meats, refined grains, and high-fat diets (≥40% energy from fat) were considered harmful.

DI-GM was calculated using dietary recall data from the NHANES 2007–2018. The components and scoring criteria for DI-GM are outlined in Supplementary Table S1. For beneficial items related to gut microbiota, a score of 1 was given when consumption exceeded the sex-specific median, and 0 otherwise. For harmful items, a score of 0 was assigned when consumption exceeded the sex-specific median or for high-fat diets (≥40% energy from fat), and a score of 1 was assigned otherwise. The total DI-GM score was derived by summing the individual item scores, yielding a range of 0 to 13 points (including beneficial components [0–9 points] and harmful components [0–4 points]). Participants were categorised into four groups based on their scores: 0–3 (Q1), 4 (Q2), 5 (Q3), and ≥6 (Q4).

### Covariates

Based on published research and clinical judgment, this study considered several potential confounders, including age, sex, ethnicity, BMI, education level, income, smoking, energy intake, physical exercise, supplement use.^(15,16)^ Total energy intake was adjusted to isolate the effect of diet composition from total caloric consumption.

Age was treated as a continuous variable in logistic regression and descriptive analyses. For subgroup analyses, age was categorised into the following groups: 20–39 years, 40–49 years, 50–59 years, 60–69 years, and ≥70 years. Race was categorised as Mexican American, non-Hispanic Black, non-Hispanic White, and other Hispanic Americans. Education level was classified as ≤ high school, high school graduate or equivalent, some college or AA degree, and college graduate or higher. Income was divided into two categories: below $20,000 and ≥$20,000. Smoking was defined as: (1) smoking >100 cigarettes, or smq020 == ‘1’; 2) smoking <100 cigarettes but currently smoking daily or occasionally. Alcohol abuse was defined as consuming >210g per week for men and >140g per week for women. Insulin resistance was assessed using HOMA-IR, calculated using the following formula: HOMA-IR = (glucose × insulin)/405.^(17)^

### Data analysis

In accordance with the NHANES analysis guidelines, this study utilised a complex sampling design and applied weights to Mobile Examination Center samples. We described the characteristics associated with MAFLD in the high-risk liver fibrosis group. New weights were constructed by dividing the 2-year MEC weights (WTMEC2YR) by 6 cycles according to NHANES analytic guidelines.

Continuous variables were presented as means with standard errors (SE), while categorical variables were reported as frequency counts and percentages (%). For the analysis of categorical data, we employed the chi-square test with Rao-Scott second-order correction. We used the Wilcoxon rank-sum test to assess the differences in continuous variables, accounting for the complex survey sampling design.

To investigate the independent associations of DI-GM with MAFLD and the risk of liver fibrosis, multivariable-weighted logistic regression models were constructed, with covariates selected based on biological plausibility and prior literature to avoid over-adjustment bias. Model 1 was unadjusted; Model 2 adjusted for age, sex, ethnicity, and total energy intake, where energy adjustment was implemented to isolate the specific effect of diet composition from total caloric consumption;^(12,18)^ Model 3 further adjusted for body mass index (BMI), socioeconomic status (education, income), lifestyle factors (smoking, physical activity), and dietary supplement use, incorporating BMI as a primary risk factor for fatty liver disease while controlling for the systemic influence of socioeconomic and lifestyle factors on metabolic health and the confounding role of exogenous nutrient supplementation.^(19-21)^

To assess the potential nonlinear relationships between DI-GM and MAFLD and the risk of liver fibrosis, we performed restricted cubic spline (RCS) analysis, defining the DI-GM range at the 10th, 50th, and 90th percentiles and fitting the curves. Moreover, we explored potential mediators of the association between DI-GM and MAFLD, such as MAF-5 score and vitamin levels. Mediation analysis was conducted using the Sobel test, bootstrap method, and quasi-Bayesian Monte Carlo method, with 1,000 simulations based on normal approximation. Given the cross-sectional nature of NHANES, the mediation analysis was performed to explore statistical associations and potential biological linkages, rather than to establish causality.

Sensitivity analyses included subgroup analyses, multiple imputation, and propensity score matching (PSM). Subgroup analyses were conducted by stratifying participants based on factors such as age, sex, ethnicity, energy, BMI, income, education level, physical exercise, smoking and supplement use. To minimise the impact of missing data on the results, multiple imputations were used to handle missing vitamin data, employing chain equations to impute missing values, and generating five imputed datasets, following the approach used in prior studies. Additionally, to further eliminate bias and control for potential confounders between the groups, PSM was conducted at a 1:1 ratio using the MAFLD and high-risk liver fibrosis groups as reference populations.^(22)^

All analyses were performed using R (version 4.2.3) and Free Software Foundation’s statistical software (version 1.9.2). All analyses accounted for the complex survey design (stratification, clustering, and weighting) using the ‘survey’ package (version 4.4.2) in R. The ‘mediation’ package (version 4.5.0) was used for mediation analysis, the ‘mice’ package (version 3.15.0) for multiple imputation, and the ‘MatchIt’ package (version 4.5.5) for PSM. Statistical significance was determined using two-tailed *p*-values < 0.05.

## Result

### Baselines

Table 1 presents the baseline characteristics of the 13,498 participants. The mean age of the entire cohort was 49.64 years (standard error 0.14). Of these, 2,327 individuals had MAFLD, and 4,268 had the high risk of liver fibrosis. The analysis revealed that individuals at a high risk for MAFLD and liver fibrosis were generally older, more likely to be male, had lower educational attainment, higher BMI, and a higher prevalence of current smoking. Additionally, these individuals exhibited higher levels of liver enzymes (e.g., AST and ALT), inflammatory markers (CRP), blood glucose levels (e.g., HbA1c, glucose, and OGTT), insulin resistance (HOMA-IR), blood pressure (BP), lower levels of HDL, and higher levels of triglycerides (TG) and total cholesterol (TCHOL). They also have higher incidence rates of metabolic disorders and diabetes.

**Table 1.** Baseline characteristics and DI-GM scores across different health groups in the NHANES 2007–2018 study

Characteristic	Overall *N* = 13,498	normal *N* = 6903	MAFLD *N* = 2327	Fibrosis *N* = 4268	*p*-value^1^
Age, Mean (SD)	49.64 (17.46)	47.29 (18.32)	50.22 (16.47)	53.13 (15.88)	<0.001
Sex, *n* (%)	–	–	–	–	<0.001
Male	6,569.00 (48.67)	2,975.00 (43.10)	943.00 (40.52)	2,651.00 (62.11)	–
Female	6,929.00 (51.33)	3,928.00 (56.90)	1,384.00 (59.48)	1,617.00 (37.89)	–
Ethnicity, *n* (%)	–	–	–	–	<0.001
White	5,695.00 (42.19)	2,907.00 (42.11)	946.00 (40.65)	1,842.00 (43.16)	–
Mexican	2,097.00 (15.54)	889.00 (12.88)	418.00 (17.96)	790.00 (18.51)	–
Black	2,687.00 (19.91)	1,345.00 (19.48)	517.00 (22.22)	825.00 (19.33)	–
Other	3,019.00 (22.37)	1,762.00 (25.53)	446.00 (19.17)	811.00 (19.00)	–
BMI, Mean (SD)	29.11 (6.78)	24.82 (3.56)	32.19 (4.34)	34.37 (7.22)	<0.001
Education level, *n* (%)	–	–	–	–	<0.001
Below high school	3,307.00 (24.50)	1,500.00 (21.73)	615.00 (26.43)	1,192.00 (27.93)	–
High school graduate/GED or equivalent	3,049.00 (22.59)	1,485.00 (21.51)	562.00 (24.15)	1,002.00 (23.48)	–
Some college or AA degree	3,930.00 (29.12)	1,955.00 (28.32)	703.00 (30.21)	1,272.00 (29.80)	–
College graduate or above	3,212.00 (23.80)	1,963.00 (28.44)	447.00 (19.21)	802.00 (18.79)	–
Income, *n* (%)	–	–	–	–	<0.001
Under $20,000	2,673.00 (20.86)	1,262.00 (19.30)	493.00 (22.53)	918.00 (22.44)	–
Over $20,000	10,144.00 (79.14)	5,277.00 (80.70)	1,695.00 (77.47)	3,172.00 (77.56)	–
Physical exercise, *n* (%)	5,129.00 (38.01)	2,606.00 (37.76)	894.00 (38.42)	1,629.00 (38.19)	0.819
Alcohol abuse, *n* (%)	410.00 (3.04)	183.00 (2.65)	76.00 (3.27)	151.00 (3.54)	0.023
Smoke, *n* (%)	6,004.00 (44.48)	2,835.00 (41.07)	1,075.00 (46.20)	2,094.00 (49.06)	<0.001
AST, Mean (SD)	25.09 (19.66)	19.34 (7.37)	20.25 (7.13)	37.04 (29.97)	<0.001
ALT, Mean (SD)	25.37 (20.71)	22.50 (7.21)	21.57 (5.95)	32.09 (34.44)	<0.001
CRP, Mean (SD)	4.18 (8.30)	2.99 (8.11)	5.51 (8.47)	5.33 (8.23)	<0.001
HAB1C, Mean (SD)	5.79 (1.10)	5.49 (0.70)	5.66 (0.81)	6.33 (1.49)	<0.001
Glucose, Mean (SD)	109.89 (35.99)	100.09 (22.41)	104.80 (25.12)	128.51 (49.26)	<0.001
OGTT Mean (SD)	121.05 (52.44)	107.01 (37.50)	120.12 (37.92)	151.50 (71.88)	<0.001
HOMA_IR, Mean (SD)	4.08 (7.17)	2.20 (2.69)	3.90 (3.85)	7.24 (11.28)	<0.001
Bpxsy, Mean (SD)	123.54 (18.47)	120.29 (18.35)	125.13 (18.04)	127.93 (17.88)	<0.001
Bpxdi, Mean (SD)	69.48 (12.77)	67.66 (12.17)	71.03 (12.41)	71.59 (13.46)	<0.001
HDL, Mean (SD)	53.84 (16.05)	59.29 (16.34)	49.40 (12.83)	47.43 (13.89)	<0.001
LDL, Mean (SD)	113.57 (35.54)	111.04 (34.39)	122.05 (35.26)	113.18 (36.85)	<0.001
TG, Mean (SD)	124.80 (101.00)	91.90 (49.04)	160.95 (115.14)	158.31 (132.54)	<0.001
TCHOL, Mean (SD)	191.86 (41.48)	188.68 (39.68)	202.75 (40.87)	191.07 (43.64)	<0.001
Metabolic_disorder, *n* (%)	9,785.00 (72.49)	3,613.00 (52.34)	2,207.00 (94.84)	3,965.00 (92.90)	<0.001
Diabetes, *n* (%)	2,892.00 (21.43)	533.00 (7.72)	185.00 (7.95)	2,174.00 (50.94)	<0.001
Energy intake, Mean (SD)	2,092.42 (985.13)	2,076.40 (975.37)	2,047.27 (985.75)	2,142.96 (998.55)	<0.001
Supplement use, *n* (%)	6,931.00 (51.35)	3,592.00 (52.04)	1,139.00 (48.95)	2,200.00 (51.55)	0.034
Alpha-carotene, Mean (SD)	5.34 (9.57)	6.83 (11.91)	4.42 (7.61)	3.38 (4.42)	<0.001
Alpha-cryptoxanthin, Mean (SD)	2.85 (1.82)	3.22 (2.13)	2.62 (1.29)	2.35 (1.36)	<0.001
Trans-beta-carotene, Mean (SD)	20.66 (24.19)	26.61 (29.83)	15.35 (13.63)	14.03 (14.37)	<0.001
Cis-beta-carotene, Mean (SD)	1.15 (1.28)	1.46 (1.62)	0.89 (0.69)	0.81 (0.64)	<0.001
Beta-cryptoxanthin, Mean (SD)	10.60 (12.13)	12.39 (14.51)	8.88 (7.43)	8.68 (9.43)	<0.001
Gamma-tocopherol, Mean (SD)	184.55 (99.89)	159.57 (76.73)	218.14 (112.29)	204.99 (114.15)	<0.001
Lutein and zeaxanthin, Mean (SD)	20.43 (14.44)	23.06 (16.88)	18.28 (10.14)	17.37 (11.16)	<0.001
Trans-lycopene, Mean (SD)	20.83 (10.89)	21.33 (11.27)	22.13 (10.96)	19.12 (9.95)	<0.001
Retinyl palmitate, Mean (SD)	1.35 (1.08)	1.37 (1.22)	1.35 (0.84)	1.33 (0.96)	0.684
Retinyl stearate, Mean (SD)	0.54 (0.29)	0.53 (0.35)	0.53 (0.14)	0.55 (0.25)	0.001
Total Lycopene, Mean (SD)	38.81 (19.72)	39.99 (20.23)	41.16 (20.56)	35.24 (17.74)	<0.001
Retinol, Mean (SD)	52.95 (16.31)	51.71 (14.91)	53.02 (15.86)	55.01 (18.54)	0.004
Alpha-tocopherol, Mean (SD)	1,215.11 (428.82)	1,180.17 (434.71)	1,273.98 (394.48)	1,235.11 (435.69)	<0.001
25-hydroxyvitamin D2, Mean (SD)	4.02 (12.03)	3.68 (11.12)	3.91 (11.29)	4.63 (13.71)	0.113
25-hydroxyvitamin D3, Mean (SD)	61.02 (27.66)	64.01 (28.70)	57.93 (25.67)	57.86 (26.43)	<0.001
Vitamin C, Mean (SD)	0.86 (0.47)	0.93 (0.46)	0.76 (0.50)	0.79 (0.45)	<0.001
Vitamin B12, Mean (SD)	618.07 (509.62)	625.15 (467.85)	586.23 (450.77)	622.60 (599.28)	<0.001
Pyridoxal 5’-phosphate, Mean (SD)	64.93 (70.22)	71.45 (76.72)	55.35 (60.96)	59.73 (62.64)	<0.001
4-pyridoxic acid, Mean (SD)	54.64 (237.60)	51.65 (188.14)	53.50 (295.46)	60.18 (270.71)	<0.001
DI_GM, Mean (SD)	4.60 (1.62)	4.71 (1.62)	4.54 (1.57)	4.46 (1.64)	<0.001

1 Kruskal–Wallis rank sum test; Pearson’s Chi-squared test.

In terms of vitamin and carotenoid levels, individuals with MAFLD and those with high-risk liver fibrosis exhibited significant differences. Overall, the levels of α-carotene, α-cryptoxanthin, trans-β-carotene, cis-β-carotene, β-cryptoxanthin, lutein, zeaxanthin, retinyl palmitate, total lycopene, and vitamin C were lower in these individuals. In contrast, the levels of γ-tocopherol, retinyl stearate, retinol, α-tocopherol, 25-hydroxyvitamin D_2_, and 4-pyridoxic acid were relatively high. Notably, compared to the normal population, individuals with MAFLD and high-risk liver fibrosis showed an inverse trend in the levels of trans-lycopene and total lycopene.

Most strikingly, the DI-GM score in individuals with MAFLD and high-risk liver fibrosis gradually decreased compared to that in the normal population. This change reflects the differences in diet and lifestyle between these groups, suggesting potential disease associations or physiological changes.

### Association between DI-GM and MAFLD and the risk of liver fibrosis

As shown in Tables 2 and 3, in unadjusted Model 1, each one-point increase in the DI-GM score was associated with an 11% reduction in the prevalence of MAFLD (OR = 0.89, 95% CI = 0.86–0.92, *p* < 0.001) and a 10% reduction in the prevalence of high-risk liver fibrosis (OR = 0.90, 95% CI = 0.87–0.93, *p* < 0.001). In fully adjusted Model 3, the association of DI-GM with MAFLD and high-risk liver fibrosis remained significant (MAFLD: OR = 0.93, 95% CI = 0.88–0.99, *p* = 0.030; high-risk liver fibrosis: OR = 0.94, 95% CI = 0.90–0.98, *p* = 0.002).

**Table 2.** Association of DI-GM with MAFLD across different models

Group	Characteristic	OR	95% CI	*p*-Value
Model1	DI_GM	0.89	0.86, 0.92	<0.001
	DI_GM_Q			
	Q1	—	—	
	Q2	0.83	0.73, 0.94	0.004
	Q3	0.74	0.65, 0.84	<0.001
	Q4	0.61	0.53, 0.70	<0.001
Model2	DI_GM	0.88	0.85, 0.91	<0.001
	DI_GM_Q			
	Q1	—	—	
	Q2	0.83	0.73, 0.95	0.006
	Q3	0.74	0.64, 0.84	<0.001
	Q4	0.59	0.51, 0.68	<0.001
Model3	DI_GM	0.93	0.88, 0.99	0.030
	DI_GM_Q			
	Q1	—	—	
	Q2	0.99	0.79, 1.24	0.912
	Q3	0.82	0.63, 1.07	0.143
	Q4	0.77	0.59, 1.00	0.050

CI = Confidence Interval, OR = Odds Ratio.

**Table 3.** Association of DI-GM with high-risk liver fibrosis across different models

Group	Characteristic	OR	95% CI	*p*-Value
Model1	DI_GM	0.90	0.87, 0.93	<0.001
	DI_GM_Q			
	Q1	—	—	
	Q2	0.85	0.73, 1.00	0.049
	Q3	0.73	0.63, 0.85	<0.001
	Q4	0.64	0.56, 0.73	<0.001
Model2	DI_GM	0.89	0.86, 0.92	<0.001
	DI_GM_Q			
	Q1	—	—	
	Q2	0.86	0.73, 1.01	0.063
	Q3	0.74	0.63, 0.86	<0.001
	Q4	0.61	0.53, 0.70	<0.001
Model3	DI_GM	0.94	0.90, 0.98	0.002
	DI_GM_Q			
	Q1	—	—	
	Q2	0.93	0.77, 1.13	0.469
	Q3	0.79	0.66, 0.95	0.015
	Q4	0.74	0.63, 0.88	<0.001

CI = Confidence Interval, OR = Odds Ratio.

When DI-GM was categorised into quartiles, in the unadjusted Model 1, participants in the highest quartile (Q4) had a significantly lower prevalence of MAFLD (OR = 0.61, 95% CI = 0.53–0.70, *p* < 0.001) and high-risk liver fibrosis (OR = 0.64, 95% CI = 0.56–0.73, *p* < 0.001) than those in the lowest quartile. Additionally, propensity score matching (PSM) was performed, and the results remained robust (Supplementary Tables S2 and S3).

### Linear and non-linear trends of DI-GM with MAFLD and the risk of liver fibrosis

**Figure 2.**
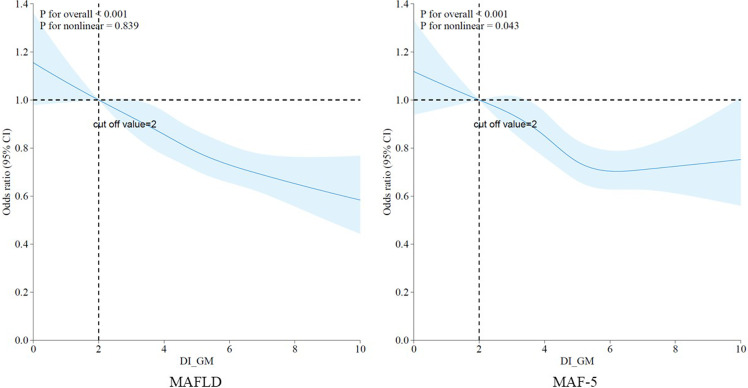
Non-linear associations of DI-GM with MAFLD and the risk of liver fibrosis.

To explore the potential non-linear relationship between the newly proposed DI-GM and MAFLD and the risk of liver fibrosis, we employed RCS in our analysis. As shown in Figure 2, after adjusting for all covariates, an overall decreasing trend in the risk of MAFLD and liver fibrosis was observed with increasing DI-GM scores (*p* < 0.001). However, the relationship between DI-GM and MAFLD was linear, with no significant non-linear association (*p* = 0.839). In contrast, a non-linear relationship was observed between DI-GM and the risk of liver fibrosis (*p* = 0.043), with a deceleration in the rate of risk reduction for both MAFLD and liver fibrosis when the DI-GM score exceeded 5.

In summary, higher DI-GM scores, which indicate a more favourable dietary pattern associated with the gut microbiota, are associated with a lower risk of MAFLD and liver fibrosis, primarily in a linear manner.

### Relationship between vitamins and MAFLD and the risk of liver fibrosis

**Figure 3.**
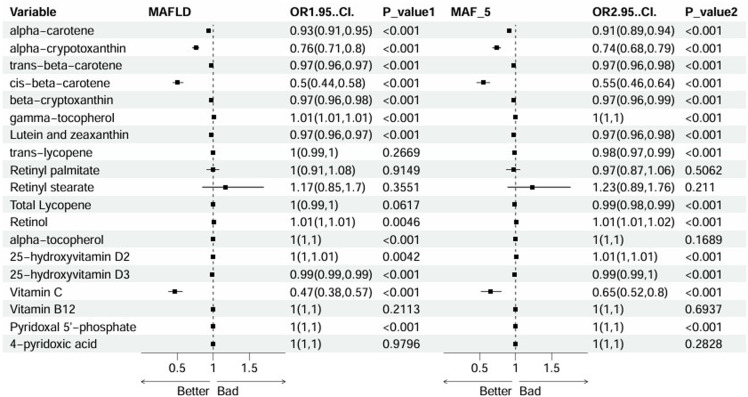
Odds ratios of vitamins and carotenoids for MAFLD and MAF-5.

To investigate the associations between various vitamins and carotenoids, MAFLD, and the risk of liver fibrosis (MAF-5), univariate logistic regression analyses were conducted. The results revealed significant associations between several vitamins and carotenoids and these diseases, suggesting their potential roles in the pathogenesis of MAFLD and liver fibrosis (Figure 3). Notably, α-carotene (MAFLD: OR = 0.93, 95% CI = 0.91–0.95, *p* < 0.001; MAF-5 ≥ 1: OR = 0.91, 95% CI = 0.89–0.94, *p* < 0.001), α-cryptoxanthin (MAFLD: OR = 0.76, 95% CI = 0.71–0.8, *p* < 0.001; MAF-5 ≥ 1: OR = 0.74, 95% CI = 0.68–0.79, *p* < 0.001), cis-β-carotene (MAFLD: OR = 0.50, 95% CI = 0.44–0.58, *p* < 0.001; MAF-5: OR = 0.55, 95% CI = 0.46–0.64, *p* < 0.001), and vitamin C (MAFLD: OR = 0.47, 95% CI = 0.38–0.57, *p* < 0.001; MAF-5: OR = 0.65, 95% CI = 0.52–0.8, *p* < 0.001) showed significant associations with both MAFLD and MAF-5.

Additionally, we used random forest imputation to address the missing values for vitamins and carotenoids in the dataset. The results were consistent with the primary findings (Supplementary Tables S4 and S5).

### Mediation analysis

To investigate the mediating effects of various vitamins and carotenoids on the relationship between the newly proposed DI-GM and MAFLD and liver fibrosis (MAF-5), we selected the four vitamins and carotenoids with the lowest odds ratios (ORs) for mediation analysis.

For MAFLD, cis-β-carotene exhibited the strongest mediating effect, accounting for 25.17% of the total effect. Additionally, the mediating effects of the other three vitamins or carotenoids (α-carotene, α-cryptoxanthin, and vitamin C) were 0.202%, 22.10%, and 22.30%, respectively.

In the MAF-5 ≥ 1 group, cis-β-carotene showed the strongest mediating effect, accounting for 63.12% of the total effect, whereas the mediating effects of the other three were 59.23% for α-carotene, 60.2% for α-cryptoxanthin, and 24.37% for vitamin C.

**Figure 4.**
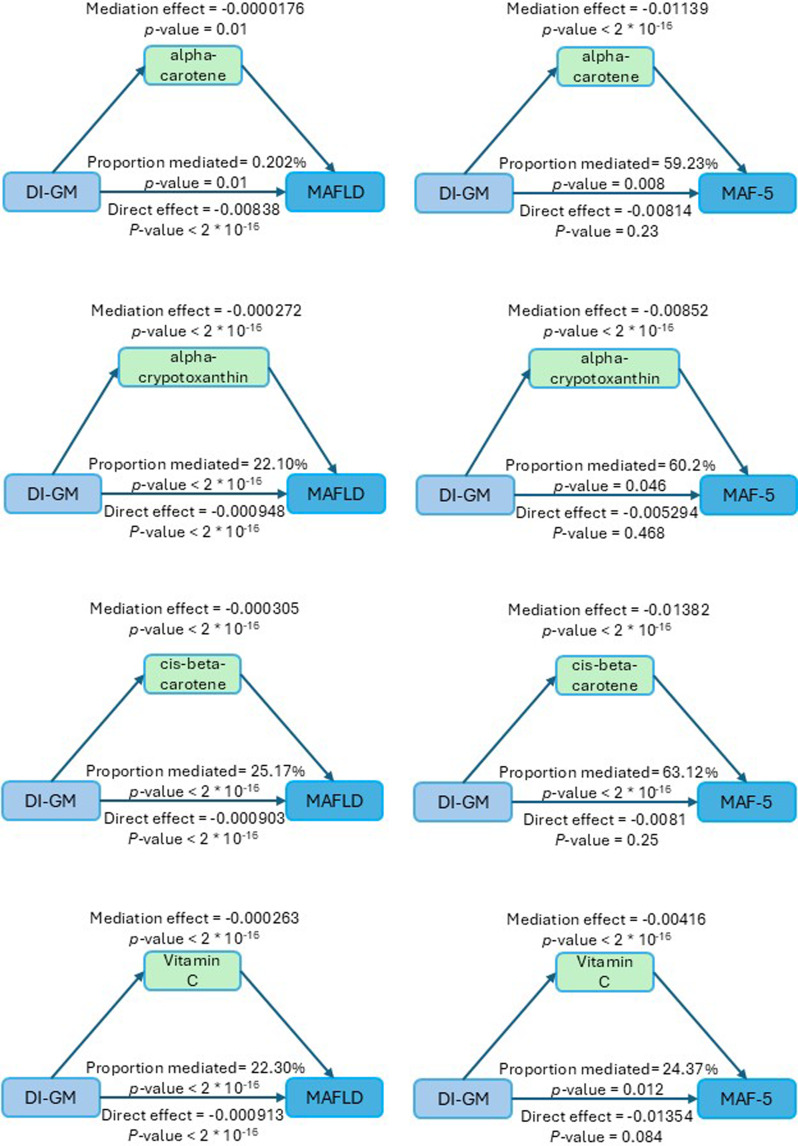
Mediation analysis of vitamins and carotenoids in the relationship between DI-GM and MAFLD/MAF-5.

These results suggest that the relationship between DI-GM, MAFLD and the risk of liver fibrosis is largely mediated by these vitamins and carotenoids, with vitamin C and cis-β-carotene playing particularly prominent roles (Figure 4).

### Subgroup analysis

To further explore the relationship between the newly proposed DI-GM and MAFLD and liver fibrosis (MAF-5), stratified analyses among the participants were conducted. As shown in Tables 4 and 5, the relationship between DI-GM and MAFLD and the risk of liver fibrosis exhibited significant heterogeneity across different subgroups.

**Table 4.** Stratified analysis of DI-GM’s association with MAFLD across demographic subgroups

	Variable	Count	Percent	OR	Lower	Upper	*P* Value	P for interaction
DI_GM	Overall	13498	100	0.91	0.89	0.93	<0.001	
	Age							0.257
	0–39	4434	32.8	0.9	0.87	0.94	<0.001	
	40–49	2289	17	0.89	0.84	0.94	<0.001	
	50–59	2280	16.9	0.86	0.82	0.91	<0.001	
	60–69	2338	17.3	0.91	0.87	0.96	<0.001	
	>= 70	2157	16	0.93	0.89	0.98	0.007	
	Sex							0.108
	Male	6569	48.7	0.93	0.91	0.96	<0.001	
	Female	6929	51.3	0.9	0.87	0.93	<0.001	
	Ethnicity							0.029
	White	5695	42.2	0.9	0.87	0.93	<0.001	
	Mexican	2097	15.5	0.99	0.93	1.05	0.71	
	Black	2687	19.9	0.92	0.88	0.97	0.002	
	Other	3019	22.4	0.9	0.85	0.94	<0.001	
	Education level							<0.001
	Below high school	3307	24.5	1	0.96	1.05	0.836	
	High school graduate/GED or Equivalent	3049	22.6	0.94	0.9	0.98	0.006	
	Some college or AA degree	3930	29.1	0.93	0.89	0.97	<0.001	
	College graduate or above	3212	23.8	0.86	0.83	0.9	<0.001	
	Income							0.203
	Under $20,000	2673	20.9	0.94	0.9	0.99	0.021	
	Over $20,000	10144	79.1	0.91	0.89	0.93	<0.001	
	Physical exercise							0.922
	No	8365	62	0.91	0.89	0.94	<0.001	
	Yes	5129	38	0.91	0.88	0.95	<0.001	
	Energy intake (Kcal/d)							0.949
	<= 1930	6755	50	0.91	0.89	0.94	<0.001	
	>1930	6743	50	0.91	0.89	0.94	<0.001	
	BMI							0.133
	<18.5	225	1.7	1	0	Inf	1	
	18.5–23.9	2799	20.7	0.76	0.62	0.93	0.007	
	24–27.9	3636	26.9	0.94	0.89	0.99	0.02	
	>28	6838	50.7	0.96	0.93	1	0.033	
	Smoke							<0.001
	No	7494	55.5	0.88	0.85	0.9	<0.001	
	Yes	6004	44.5	0.96	0.93	0.99	0.021	
	Supplement use							0.196
	No	6566	48.6	0.93	0.9	0.96	<0.001	
	Yes	6931	51.4	0.9	0.88	0.93	<0.001	

**Table 5.** Stratified analysis of DI-GM’s association with liver fibrosis (MAF-5) across demographic subgroups

	Variable	Count	Percent	OR	Lower	Upper	*p* Value	*P* for interaction
DI_GM	Overall	13498	100	0.92	0.9	0.94	<0.001	
	Age							0.197
	0–39	4434	32.8	0.91	0.87	0.96	<0.001	
	40–49	2289	17	0.86	0.81	0.91	<0.001	
	50–59	2280	16.9	0.88	0.84	0.93	<0.001	
	60–69	2338	17.3	0.93	0.89	0.98	0.004	
	>= 70	2157	16	0.93	0.88	0.98	0.009	
	Sex							0.17
	Male	6569	48.7	0.95	0.92	0.97	<0.001	
	Female	6929	51.3	0.92	0.88	0.95	<0.001	
	Ethnicity							0.167
	White	5695	42.2	0.9	0.87	0.93	<0.001	
	Mexican	2097	15.5	0.97	0.92	1.03	0.331	
	Black	2687	19.9	0.94	0.89	0.99	0.023	
	Other	3019	22.4	0.92	0.87	0.96	0.001	
	Education level							0.034
	Below high school	3307	24.5	0.98	0.93	1.02	0.342	
	High school graduate/GED or Equivalent	3049	22.6	0.96	0.92	1.01	0.117	
	Some college or AA degree	3930	29.1	0.92	0.88	0.96	<0.001	
	College graduate or above	3212	23.8	0.89	0.85	0.94	<0.001	
	Income							0.174
	Under $20,000	2673	20.9	0.96	0.91	1.01	0.097	
	Over $20,000	10144	79.1	0.92	0.9	0.94	<0.001	
	Physical exercise							0.168
	No	8365	62	0.91	0.89	0.94	<0.001	
	Yes	5129	38	0.94	0.91	0.98	0.001	
	Energy intake (Kcal/d)							0.927
	<= 1930	6755	50	0.92	0.89	0.95	<0.001	
	>1930	6743	50	0.92	0.89	0.95	<0.001	
	BMI							0.412
	<18.5	225	1.7	0.63	0.34	1.16	0.136	
	18.5–23.9	2799	20.7	0.91	0.82	1.01	0.068	
	24–27.9	3636	26.9	0.96	0.91	1.01	0.11	
	>28	6838	50.7	0.96	0.93	0.99	0.006	
	Smoke							0.001
	No	7494	55.5	0.89	0.86	0.92	<0.001	
	Yes	6004	44.5	0.96	0.93	1	0.024	
	Supplement use							0.432
	No	6566	48.6	0.93	0.9	0.96	<0.001	
	Yes	6931	51.4	0.91	0.89	0.94	<0.001	

First, regarding the relationship between DI-GM and MAFLD, significant interactions were observed between age, sex, education level, and smoking status. In the 50–59 age group, the association between DI-GM and MAFLD was most pronounced (OR = 0.86, 95% CI = 0.82–0.91, *p* < 0.001). Regarding sex, the negative correlation between DI-GM and MAFLD was stronger in females (OR = 0.90, 95% CI = 0.87–0.93, *p* < 0.001). Additionally, individuals with a college degree or higher also exhibited a stronger protective effect against DI-GM (OR = 0.86, 95% CI = 0.83–0.90, *p* < 0.001). These findings suggest that education level may play a moderating role in the association between DI-GM and MAFLD, potentially related to the dissemination of health knowledge and lifestyle improvements.

Smoking status also exhibited a significant interaction with the relationship between DI-GM scores and MAFLD. Among smokers, the association between DI-GM and MAFLD was more pronounced (OR = 0.96, 95% CI = 0.93–0.99, *p* = 0.023), suggesting that smoking may enhance the protective effect of DI-GM against MAFLD. In contrast, among non-smokers, the relationship between DI-GM and MAFLD was weaker (OR = 0.88, 95% CI = 0.85–0.90, *p* < 0.001). This may be related to the potential negative impacts of smoking on the gut microbiota and metabolic function, where the detrimental effects of smoking might amplify the protective effect of DI-GM.

Regarding the relationship between DI-GM and the risk of liver fibrosis, significant interactions were also observed between age and sex. The protective effect of DI-GM was particularly strong in the 40–49 age group (OR = 0.86, 95% CI = 0.81–0.91, *p* < 0.001) and among males (OR = 0.95, 95% CI = 0.92–0.97, *p* < 0.001). Additionally, smoking status was associated with the relationship between DI-GM and the risk of liver fibrosis. Compared to non-smokers, the negative correlation between DI-GM and the risk of liver fibrosis was stronger among smokers (OR = 0.96, 95% CI = 0.93–1.00, *p* = 0.024), indicating that smoking may further enhance the protective effect of DI-GM against liver fibrosis through its impact on hepatic metabolic function.

### Supplementation needs for low DI-GM in MAFLD and high-risk fibrosis populations

To further explore the clinical implications of the DI-GM scores, we analysed the levels of vitamins and carotenoids among individuals at a high risk for MAFLD and liver fibrosis. As shown in Tables 6 and 7, individuals in the low DI-GM group with high-risk MAFLD and liver fibrosis exhibited significantly lower concentrations of most vitamins and carotenoids, particularly in Q1 and Q2. Specifically, in the high-risk MAFLD group, the levels of the majority of vitamins and carotenoids (such as α-carotene, β-cryptoxanthin, lutein, zeaxanthin and vitamin C.) were significantly higher in the Q3 and Q4 groups, with the exception of a few vitamins (e.g., γ-tocopherol). This suggests that intake of these nutrients may be associated with higher DI-GM scores, further supporting DI-GM as a potential modulator of metabolic diseases.

**Table 6.** Vitamin and carotenoid levels in high-risk MAFLD groups by DI-GM quartiles

Characteristic	Overall *N* = 5,940	Q1 *N* = 1,613	Q2 *N* = 1,520	Q3 *N* = 1,327	Q4 *N* = 1,480	*p*-Value
Alpha-carotene, Mean (SD)	3.75 (6.02)	2.84 (3.40)	3.46 (7.77)	4.07 (5.80)	4.77 (6.26)	<0.001
Alpha-cryptoxanthin, Mean (SD)	2.46 (1.33)	2.23 (1.14)	2.38 (1.32)	2.56 (1.34)	2.71 (1.48)	<0.001
Trans-beta-carotene, Mean (SD)	14.10 (13.57)	11.19 (11.37)	12.99 (12.56)	15.02 (13.43)	17.68 (15.83)	<0.001
Cis-beta-carotene, Mean (SD)	0.82 (0.64)	0.70 (0.53)	0.77 (0.62)	0.87 (0.68)	0.96 (0.70)	<0.001
Beta-cryptoxanthin, Mean (SD)	8.52 (8.10)	6.99 (6.51)	7.79 (7.70)	9.29 (7.18)	10.33 (10.10)	<0.001
Gamma-tocopherol, Mean (SD)	214.58 (114.76)	225.43 (116.90)	225.06 (128.96)	204.54 (109.29)	199.75 (99.08)	0.022
Lutein and zeaxanthin, Mean (SD)	17.37 (9.96)	14.67 (7.65)	16.26 (8.92)	18.12 (10.65)	20.80 (11.46)	<0.001
Trans-lycopene, Mean (SD)	20.55 (10.46)	20.48 (10.31)	20.04 (10.46)	21.10 (10.80)	20.71 (10.39)	0.808
Retinyl palmitate, Mean (SD)	1.35 (0.94)	1.30 (0.93)	1.36 (1.10)	1.32 (0.79)	1.41 (0.88)	0.047
Retinyl stearate, Mean (SD)	0.54 (0.22)	0.54 (0.29)	0.54 (0.19)	0.55 (0.17)	0.54 (0.20)	0.291
Total Lycopene, Mean (SD)	37.92 (19.19)	37.46 (19.23)	37.13 (19.00)	39.43 (20.46)	38.03 (18.31)	0.696
Retinol, Mean (SD)	54.04 (17.57)	53.76 (18.68)	52.93 (17.03)	52.93 (17.36)	56.28 (16.83)	0.035
Alpha-tocopherol, Mean (SD)	1,254.46 (415.48)	1,162.76 (370.53)	1,227.44 (434.24)	1,299.26 (420.36)	1,347.50 (418.61)	<0.001
Pyridoxal 5’-phosphate, Mean (SD)	56.41 (60.80)	51.98 (56.12)	50.07 (48.70)	55.83 (59.84)	67.50 (73.83)	<0.001
Retinyl stearate.1, Mean (SD)	0.54 (0.22)	0.54 (0.29)	0.54 (0.19)	0.55 (0.17)	0.54 (0.20)	0.291
25-hydroxyvitamin D2, Mean (SD)	4.34 (12.78)	4.30 (13.00)	4.47 (13.14)	4.05 (12.23)	4.51 (12.63)	<0.001
25-hydroxyvitamin D3, Mean (SD)	57.32 (25.84)	54.97 (26.21)	55.81 (25.35)	57.78 (25.56)	61.02 (25.79)	<0.001
Vitamin C, Mean (SD)	0.77 (0.46)	0.66 (0.39)	0.75 (0.44)	0.80 (0.45)	0.89 (0.53)	<0.001
Vitamin B12, Mean (SD)	606.86 (571.00)	561.95 (405.36)	617.01 (764.92)	591.15 (378.11)	656.07 (601.34)	0.008
Pyridoxal 5’-phosphate.1, Mean (SD)	56.41 (60.80)	51.98 (56.12)	50.07 (48.70)	55.83 (59.84)	67.50 (73.83)	<0.001
4-pyridoxic acid, Mean (SD)	54.57 (257.48)	49.18 (331.28)	54.34 (256.19)	41.84 (66.03)	71.97 (277.98)	<0.001

Kruskal–Wallis rank sum test.

**Table 7.** Vitamin and carotenoid levels in high-risk liver fibrosis groups by DI-GM quartiles

Characteristic	Overall *N* = 4,268	Q1 *N* = 1,171	Q2 *N* = 1,115	Q3 *N* = 925	Q4 *N* = 1,057	*p*-Value
Alpha-carotene, Mean (SD)	3.38 (4.42)	2.93 (3.86)	2.92 (3.17)	3.68 (3.40)	4.20 (6.41)	<0.001
Alpha-cryptoxanthin, Mean (SD)	2.35 (1.36)	2.18 (1.23)	2.27 (1.33)	2.53 (1.49)	2.52 (1.42)	0.024
Trans-beta-carotene, Mean (SD)	14.03 (14.37)	11.61 (13.31)	13.65 (14.77)	15.46 (13.43)	16.18 (15.39)	<0.001
Cis-beta-carotene, Mean (SD)	0.81 (0.64)	0.71 (0.58)	0.81 (0.71)	0.88 (0.64)	0.88 (0.61)	<0.001
Beta-cryptoxanthin, Mean (SD)	8.68 (9.43)	7.07 (7.14)	7.81 (9.71)	10.24 (9.21)	10.35 (11.05)	<0.001
Gamma-tocopherol, Mean (SD)	204.99 (114.15)	217.87 (122.92)	214.97 (136.97)	184.23 (85.26)	193.33 (87.75)	0.143
Lutein and zeaxanthin, Mean (SD)	17.37 (11.16)	15.43 (11.11)	16.12 (9.55)	18.97 (12.83)	19.80 (11.11)	<0.001
Trans-lycopene, Mean (SD)	19.12 (9.95)	19.93 (10.04)	18.63 (9.94)	19.12 (10.58)	18.69 (9.38)	0.612
Retinyl palmitate, Mean (SD)	1.33 (0.96)	1.39 (1.05)	1.40 (1.18)	1.18 (0.58)	1.28 (0.77)	0.721
Retinyl stearate, Mean (SD)	0.55 (0.25)	0.56 (0.34)	0.56 (0.21)	0.54 (0.16)	0.54 (0.21)	0.266
Total Lycopene, Mean (SD)	35.24 (17.74)	36.34 (17.34)	34.41 (17.57)	36.00 (20.39)	34.34 (16.33)	0.7
Retinol, Mean (SD)	55.01 (18.54)	54.80 (20.39)	53.65 (17.79)	55.44 (18.41)	56.49 (17.19)	0.273
Alpha-tocopherol, Mean (SD)	1,235.11 (435.69)	1,178.43 (439.22)	1,251.98 (494.80)	1,270.73 (414.09)	1,255.00 (369.39)	0.045
Pyridoxal 5’-phosphate, Mean (SD)	59.73 (62.64)	54.03 (57.22)	55.83 (56.09)	60.84 (65.35)	68.85 (70.77)	<0.001
Retinyl stearate.1, Mean (SD)	0.55 (0.25)	0.56 (0.34)	0.56 (0.21)	0.54 (0.16)	0.54 (0.21)	0.266
25-hydroxyvitamin D2, Mean (SD)	4.63 (13.71)	4.37 (13.09)	5.16 (15.41)	4.44 (12.89)	4.55 (13.20)	<0.001
25-hydroxyvitamin D3, Mean (SD)	57.86 (26.43)	55.23 (26.44)	56.46 (26.71)	59.28 (26.56)	61.01 (25.63)	<0.001
Vitamin C, Mean (SD)	0.79 (0.45)	0.71 (0.40)	0.77 (0.48)	0.83 (0.46)	0.88 (0.45)	<0.001
Vitamin B12, Mean (SD)	622.60 (599.28)	558.92 (410.71)	634.89 (791.41)	607.18 (405.39)	690.05 (667.53)	0.004
Pyridoxal 5’-phosphate.1, Mean (SD)	59.73 (62.64)	54.03 (57.22)	55.83 (56.09)	60.84 (65.35)	68.85 (70.77)	<0.001
4-pyridoxic acid, Mean (SD)	60.18 (270.71)	40.01 (109.58)	71.61 (348.30)	62.70 (299.25)	67.88 (274.96)	<0.001

Kruskal–Wallis rank sum test.

The distribution of vitamin and carotenoid levels in the high-risk liver fibrosis group was similar to that in the MAFLD group. Individuals in the Q3 and Q4 groups generally exhibited higher concentrations of carotenoids (such as α-carotene, trans-β-carotene, and β-cryptoxanthin) and vitamins (such as vitamin C, vitamin B12, and vitamin D3). Notably, the γ-tocopherol levels were higher in the Q1 group, with minimal differences observed among the other groups (Table 7). These findings suggest that individuals with low DI-GM scores may experience deficiencies in certain nutrients, particularly carotenoids and vitamins, further highlighting the necessity of supplementing high-risk populations with these nutrients.

Overall, the associations between DI-GM and MAFLD and liver fibrosis not only reflect the potential role of gut microbiota in metabolic diseases, but also provide new insights for clinical guidance on nutritional supplementation strategies involving vitamins and carotenoids. Specifically, in high-risk populations with low DI-GM scores, supplementation with relevant vitamins and carotenoids may help alleviate the metabolic liver burden and improve overall health status.

### Sensitivity analysis

To verify the consistency and robustness of the results, we conducted multiple sensitivity analyses. We further confirmed the robustness of our findings using PSM, random forest imputation, and subgroup analyses. Additionally, to exclude potential confounding factors, we performed analyses excluding individuals aged 75 years or older and those with supplement use in fully adjusted Model 3. The results showed that the associations between DI-GM, MAFLD and the risk of liver fibrosis remained unchanged after excluding these specific groups. This further supports the robustness of our primary analysis results, indicating that the effects of DI-GM are consistent across different populations and are not significantly influenced by age or supplement use (Supplementary Tables S6 and S7).

## Discussion

This study is the first to investigate the relationship between DI-GM and MAFLD and the risk of liver fibrosis, as well as the mediating roles of vitamins and carotenoids. The results demonstrate that higher DI-GM scores are associated with a reduced prevalence of MAFLD and high-risk liver fibrosis, and these associations are largely mediated by specific vitamins and carotenoids. This finding offers new insights into the prevention and management of MAFLD and liver fibrosis, particularly through dietary interventions targeting the gut microbiota and the potential supplementation of relevant nutrients.

The influence of diet on the gut microbiota and its potential role in the pathogenesis of MAFLD and liver fibrosis have been a major focus of research.^(23,24)^ In this study, DI-GM reflected dietary patterns that were either beneficial or detrimental to gut health, and its variations were associated with the risk of MAFLD and liver fibrosis.

For example, foods rich in dietary fibre, such as whole grains, legumes, vegetables, and fruits, are considered beneficial components of DI-GM. These foods promote the production of short-chain fatty acids such as propionate and butyrate by gut microbiota, which are beneficial for gut health.^(25)^ Additionally, the mediterranean diet has been shown to have positive effects on the gut microbiota and human health, potentially aiding in the prevention and mitigation of MAFLD and liver fibrosis progression.^(26)^

In contrast, refined grains and diets that are high in fat and carbohydrates are considered unfavourable components of DI-GM. These dietary habits not only alter the composition of the gut microbiota but may also exacerbate the progression of MAFLD and liver fibrosis by increasing inflammatory responses and reducing the production of beneficial metabolites.^(27,28)^

Reduced gut microbiota diversity is closely associated with an increased risk of MAFLD and liver fibrosis.^(29)^ Patients with MAFLD typically exhibit decreased α-diversity of the gut microbiota, along with an increase in harmful bacterial taxa and a reduction in beneficial bacterial taxa. This reduction in gut microbiota diversity may impact liver health through the gut–microbiota–liver axis.

Unhealthy lifestyle habits, excessive use of antibiotics, and chronic inflammation can alter the structure of the gut barrier, increasing the intestinal permeability to bacteria and their metabolites. As a result, bacterial products can enter the bloodstream, exerting direct toxic effects on the liver, further inducing hepatic inflammation, and accelerating the progression of MAFLD and liver fibrosis. Additionally, endotoxins from the gut, such as lipopolysaccharide (LPS), can enter the intestinal capillaries and activate Toll-like receptor (TLR) signalling pathways, triggering hepatic inflammation and fibrosis and promoting inflammatory responses and insulin resistance.^(30,31)^ The high mediation likely reflects the strong biological coupling between this specific dietary pattern, improved gut absorption, and the resulting systemic antioxidant status, rather than a single isolated mechanistic pathway.

Vitamins and carotenoids play a significant role in the progression of MAFLD, and liver fibrosis is influenced by gut microbiota.^(32,33)^ In this study, α-carotene, α-cryptoxanthin, cis-β-carotene, and vitamin C were identified as significant mediators of the association between DI-GM, MAFLD, and liver fibrosis. These vitamins and carotenoids, which act as antioxidants, can reduce oxidative stress and protect hepatocytes from damage.

Metabolites produced by the gut microbiota, such as short-chain fatty acids, can modulate the host’s antioxidant defence mechanisms and work synergistically with vitamins C and E to enhance antioxidant effects. Additionally, certain gut bacteria are capable of synthesising or transforming vitamins, such as vitamins K and B. These vitamins play important roles in regulating liver metabolism and immune function, which may help mitigate MAFLD progression.^(31)^

Despite the valuable insights provided by this study, several limitations of this study should be acknowledged. First, its cross-sectional design restricts the determination of causality. Future prospective studies and randomised controlled trials are needed to validate the causal relationships between DI-GM and MAFLD and the risk of liver fibrosis. Second, although we controlled for multiple potential confounders, the results may still be subject to bias owing to unmeasured variables or unknown confounding effects. Third, the DI-GM was constructed based on 14 food items; however, the NHANES 24-hour dietary recall data did not capture the consumption of specific types of tea, and thus, the relevant food parameters were not included in the analysis. This omission may have affected the comprehensiveness of the model and potentially underestimated the effects of certain dietary components. Lastly, self-reported 24-hour dietary records may introduce recall bias, and some covariates were also based on self-reports, which may affect the accuracy of the data.

Future research could address these limitations by using proxy variables (e.g., other antioxidant-rich teas) to supplement the missing green tea data, or by collecting more detailed dietary information to improve the integrity of the DI-GM model and enhance the accuracy of the analysis.

## Conclusion

In summary, this study revealed significant associations between DI-GM, MAFLD and the risk of liver fibrosis, highlighting the important mediating roles of vitamins and carotenoids. These findings provide new insights into the prevention and management of MAFLD and its complications through dietary interventions targeting the gut microbiota and supplementation of relevant nutrients. Future research should explore the causal mechanisms underlying these associations and validate the effectiveness of these interventions.

## Supporting information

10.1017/jns.2026.10093.sm001 Han et al. supplementary material 1 Han et al. supplementary material

10.1017/jns.2026.10093.sm002 Han et al. supplementary material 2 Han et al. supplementary material

10.1017/jns.2026.10093.sm003 Han et al. supplementary material 3 Han et al. supplementary material

10.1017/jns.2026.10093.sm004 Han et al. supplementary material 4 Han et al. supplementary material

10.1017/jns.2026.10093.sm005 Han et al. supplementary material 5 Han et al. supplementary material

10.1017/jns.2026.10093.sm006 Han et al. supplementary material 6 Han et al. supplementary material

10.1017/jns.2026.10093.sm007 Han et al. supplementary material 7 Han et al. supplementary material

10.1017/jns.2026.10093.sm008 Han et al. supplementary material 8 Han et al. supplementary material

## Data Availability

Data can be downloaded from the NHANES website (https://wwwn.cdc.gov/nchs/nhanes/default.aspx).

## Supplementary material

The supplementary material for this article can be found at https://doi.org/10.1017/jns.2026.10093.
