# Supplementary material for: Association of gut microbiota dietary index with MAFLD and the risk of liver fibrosis: the mediating effect of vitamins
Source: J Nutr Sci. 2026 Apr 13;15:e23. doi: 10.1017/jns.2026.10093 (PMC13126062; doi:10.1017/jns.2026.10093)
Supplement: Han et al. supplementary material 1 — Han et al. supplementary material [file S2048679026100937sup001.zip › Supplementary Materials/Supplementary Table S2.docx]

Supplementary Table S2：Odds Ratios of MAFLD by Quartiles of DI-GM in NHANES 2007-2018

| **Group** | **Characteristic** | **OR** | **95% CI** | **p-value** |
| --- | --- | --- | --- | --- |
| Model1 | DI_GM | 0.90 | 0.87, 0.93 | <0.001 |
|  | DI_GM_Q |  |  |  |
|  | Q1 | — | — |  |
|  | Q2 | 0.84 | 0.73, 0.97 | 0.015 |
|  | Q3 | 0.76 | 0.66, 0.88 | <0.001 |
|  | Q4 | 0.64 | 0.56, 0.74 | <0.001 |
| Model2 | DI_GM | 0.89 | 0.86, 0.92 | <0.001 |
|  | DI_GM_Q |  |  |  |
|  | Q1 | — | — |  |
|  | Q2 | 0.84 | 0.73, 0.96 | 0.014 |
|  | Q3 | 0.76 | 0.65, 0.88 | <0.001 |
|  | Q4 | 0.62 | 0.54, 0.72 | <0.001 |
| Model3 | DI_GM | 0.93 | 0.88, 0.99 | 0.031 |
|  | DI_GM_Q |  |  |  |
|  | Q1 | — | — |  |
|  | Q2 | 0.99 | 0.79, 1.24 | 0.906 |
|  | Q3 | 0.82 | 0.64, 1.07 | 0.141 |
|  | Q4 | 0.77 | 0.59, 1.00 | 0.050 |
| Abbreviations: CI = Confidence Interval, OR = Odds Ratio | | | | |
