# Supplementary material for: Association of gut microbiota dietary index with MAFLD and the risk of liver fibrosis: the mediating effect of vitamins
Source: J Nutr Sci. 2026 Apr 13;15:e23. doi: 10.1017/jns.2026.10093 (PMC13126062; doi:10.1017/jns.2026.10093)
Supplement: Han et al. supplementary material 1 — Han et al. supplementary material [file S2048679026100937sup001.zip › Supplementary Materials/Supplementary Table S3.docx]

Supplementary Table S3: Odds Ratios of High-Risk Liver Fibrosis by Quartiles of DI-GM in NHANES 2007-2018

| **Group** | **Characteristic** | **OR** | **95% CI** | **p-value** |
| --- | --- | --- | --- | --- |
| Model1 | DI_GM | 0.93 | 0.89, 0.97 | <0.001 |
|  | DI_GM_Q |  |  |  |
|  | Q1 | — | — |  |
|  | Q2 | 0.93 | 0.78, 1.11 | 0.435 |
|  | Q3 | 0.80 | 0.67, 0.96 | 0.015 |
|  | Q4 | 0.72 | 0.61, 0.85 | <0.001 |
| Model2 | DI_GM | 0.92 | 0.89, 0.96 | <0.001 |
|  | DI_GM_Q |  |  |  |
|  | Q1 | — | — |  |
|  | Q2 | 0.93 | 0.78, 1.11 | 0.437 |
|  | Q3 | 0.80 | 0.67, 0.95 | 0.012 |
|  | Q4 | 0.70 | 0.59, 0.83 | <0.001 |
| Model3 | DI_GM | 0.94 | 0.90, 0.98 | 0.004 |
|  | DI_GM_Q |  |  |  |
|  | Q1 | — | — |  |
|  | Q2 | 0.96 | 0.79, 1.16 | 0.677 |
|  | Q3 | 0.81 | 0.67, 0.98 | 0.029 |
|  | Q4 | 0.76 | 0.64, 0.91 | 0.003 |
| Abbreviations: CI = Confidence Interval, OR = Odds Ratio | | | | |
