# Supplementary material for: Association of gut microbiota dietary index with MAFLD and the risk of liver fibrosis: the mediating effect of vitamins
Source: J Nutr Sci. 2026 Apr 13;15:e23. doi: 10.1017/jns.2026.10093 (PMC13126062; doi:10.1017/jns.2026.10093)
Supplement: Han et al. supplementary material 1 — Han et al. supplementary material [file S2048679026100937sup001.zip › Supplementary Materials/Supplementary Table S4.docx]

Supplementary Table S4：Associations of Vitamins and Carotenoids with MAFLD Using Random Forest Imputation

| Variable | OR | Lower_CI | Upper_CI | P_value |
| --- | --- | --- | --- | --- |
| Alpha_carotene | 0.99 | 0.99 | 0.99 | <0.001 |
| Alpha_crypotoxanthin | 0.88 | 0.86 | 0.9 | <0.001 |
| Trans_beta_carotene | 0.99 | 0.99 | 0.99 | <0.001 |
| Cis_beta_carotene | 0.8 | 0.77 | 0.83 | <0.001 |
| Beta_cryptoxanthin | 0.98 | 0.98 | 0.99 | <0.001 |
| Gamma_tocopherol | 1.01 | 1.01 | 1.01 | <0.001 |
| Lutein_and_zeaxanthin | 0.98 | 0.98 | 0.98 | <0.001 |
| Trans_lycopene_ | 0.99 | 0.99 | 1 | 0.002 |
| Retinyl_palmitate | 1.07 | 1.02 | 1.12 | 0.0031 |
| Retinyl_stearate | 1.41 | 1.18 | 1.71 | <0.001 |
| Total_Lycopene | 1 | 1 | 1 | 0.0322 |
| Retinol | 1.01 | 1.01 | 1.01 | <0.001 |
| alpha_tocopherol | 1 | 1 | 1 | <0.001 |
| X25_hydroxyvitamin_D2 | 1 | 1 | 1.01 | 0.005 |
| X25_hydroxyvitamin_D3 | 0.99 | 0.99 | 0.99 | <0.001 |
| Vitamin_C_ | 0.49 | 0.46 | 0.53 | <0.001 |
| Vitamin_B12 | 1 | 1 | 1 | <0.001 |
| Pyridoxal_5_phosphate | 1 | 1 | 1 | <0.001 |
| X4_pyridoxic_acid_ | 1 | 1 | 1 | 0.0374 |
