# Supplementary material for: Association of gut microbiota dietary index with MAFLD and the risk of liver fibrosis: the mediating effect of vitamins
Source: J Nutr Sci. 2026 Apr 13;15:e23. doi: 10.1017/jns.2026.10093 (PMC13126062; doi:10.1017/jns.2026.10093)
Supplement: Han et al. supplementary material 1 — Han et al. supplementary material [file S2048679026100937sup001.zip › Supplementary Materials/Supplementary Table S6.docx]

Supplementary Table S6: Sensitivity Analysis of DI-GM's Association with MAFLD and Liver Fibrosis (MAF-5) Excluding Older Adults (Age >= 75)

|  | MAFLD | | | MAF-5 | | |
| --- | --- | --- | --- | --- | --- | --- |
| **Characteristic** | **OR** | **95% CI** | **p-value** | **OR** | **95% CI** | **p-value** |
| DI_GM | 0.92 | 0.86, 0.98 | 0.012 | 0.92 | 0.88, 0.97 | <0.001 |
| DI_GM_Q |  |  |  |  |  |  |
| Q1 | — | — |  | — | — |  |
| Q2 | 0.99 | 0.78, 1.26 | 0.952 | 0.90 | 0.73, 1.11 | 0.304 |
| Q3 | 0.81 | 0.61, 1.07 | 0.141 | 0.78 | 0.63, 0.95 | 0.015 |
| Q4 | 0.72 | 0.54, 0.95 | 0.021 | 0.69 | 0.57, 0.84 | <0.001 |
| Abbreviations: CI = Confidence Interval, OR = Odds Ratio | | | | | | |
