# Supplementary material for: Association of gut microbiota dietary index with MAFLD and the risk of liver fibrosis: the mediating effect of vitamins
Source: J Nutr Sci. 2026 Apr 13;15:e23. doi: 10.1017/jns.2026.10093 (PMC13126062; doi:10.1017/jns.2026.10093)
Supplement: Han et al. supplementary material 1 — Han et al. supplementary material [file S2048679026100937sup001.zip › Supplementary Materials/Supplementary Table S7.docx]

Supplementary Table S7: Sensitivity Analysis of DI-GM's Association with MAFLD and Liver Fibrosis (MAF-5) Excluding Individuals with Supplement Use

|  | MAFLD | | | MAF-5 | | |
| --- | --- | --- | --- | --- | --- | --- |
| **Characteristic** | **OR** | **95% CI** | **p-value** | **OR** | **95% CI** | **p-value** |
| DI_GM | 0.93 | 0.87, 0.99 | 0.022 | 0.94 | 0.91, 0.98 | 0.004 |
| DI_GM_Q |  |  |  |  |  |  |
| Q1 | — | — |  | — | — |  |
| Q2 | 0.99 | 0.79, 1.24 | 0.902 | 0.93 | 0.77, 1.14 | 0.485 |
| Q3 | 0.81 | 0.63, 1.05 | 0.118 | 0.80 | 0.66, 0.97 | 0.022 |
| Q4 | 0.76 | 0.58, 0.98 | 0.037 | 0.76 | 0.64, 0.90 | 0.002 |
| Abbreviations: CI = Confidence Interval, OR = Odds Ratio | | | | | | |
